# Engineering Composites Made from Wood and Chicken Feather Bonded with UF Resin Fortified with Wollastonite: A Novel Approach

**DOI:** 10.3390/polym12040857

**Published:** 2020-04-07

**Authors:** Hamid R. Taghiyari, Roya Majidi, Ayoub Esmailpour, Younes Sarvari Samadi, Asghar Jahangiri, Antonios N. Papadopoulos

**Affiliations:** 1 Wood Science and Technology Department, Faculty of Materials Engineering and New Technologies, Shahid Rajaee Teacher Training University, Tehran 1678815811, Iran; mohamad.mj8@yahoo.com; 2 Department of Physics, Faculty of Sciences, Shahid Rajaee Teacher Training University, Tehran 1678815811, Iran; r.majidi@sru.ac.ir (R.M.); esmailpour@sru.ac.ir (A.E.); 3 Faculty of Wood Technology and Construction, Rosenheim University of Applied Sciences, Rosenheim 83024, Germany; younes.sarvari@gmail.com; 4 Laboratory of Wood Chemistry and Technology, Department of Forestry and Natural Environment, International Hellenic University, GR-661 00 Drama, Greece

**Keywords:** engineering materials, composite panels, chicken feather, cell-wall polymers, thermal conductivity coefficient, wollastonite, wood, natural materials

## Abstract

Wood-composite panel factories are in shortage of raw materials; therefore, finding new sources of fibers is vital for sustainable production. The effects of chicken feathers, as a renewable source of natural fibers, on the physicomechanical properties of medium-density fiberboard (MDF) and particleboard panels were investigated here. Wollastonite was added to resin to compensate possible negative effects of chicken feathers. Only feathers of the bodies of chickens were added to composite matrix at 5% and 10% content, based on the dry weight of the raw material, particles or fibers. Results showed significant negative effects of 10%-feather content on physical and mechanical properties. However, feather content of 5% showed some promising results. Addition of wollastonite to resin resulted in the improvement of some physical and mechanical properties. Wollastonite acted as reinforcing filler in resin and improved some of the properties; therefore, future studies should be carried out on the reduction of resin content. Moreover, density functional theory (DFT) demonstrated the formation of new bonds between wollastonite and carbohydrate polymers in the wood cell wall. It was concluded that chicken feathers have potential in wood-composite panel production.

## 1. Introduction

Fast-growing wood species are often used in the manufacture of composite panels, engineered and modified wood, and paper industries [1,2]. Therefore, their use is of advantage since they offer a homogeneous structure which is of great importance for many general and specific purposes [3,4,5]. Composite manufacturing factories have always been confronted with some ongoing issues, such as the emission of formaldehyde, heat transfer to the core of the mat, vulnerability to vapor, and biological susceptibility to fungi and insects [6,7,8,9,10]. Moreover, numerous studies have been focused on the limitation of formaldehyde emission and on the improvement of the resin bond [11]. The heat-transferring properties of metals and improving effects of different materials at micro- and nano-scales [12,13,14,15,16,17] were also found to decrease hot press time and to improve the physicomechanical properties in wood composites [6,18]. Under this frame, wollastonite (as a silicate mineral, CaSiO_3_) was found to improve the biological and physicomechanical properties of both solid wood and wood based panels, as well as to improve the fire retardancy and to increase thermal conductivity coefficient in medium-density fiberboards (MDF) [19,20,21,22,23,24,25,26], therefore, the first aim of the present study was to find out possible effects that wollastonite may have on physical and mechanical properties of two engineering wood composites, namely medium-density fiberboards and particleboards. Based on potential positive results of the addition of wollastonite on properties of composite panels in the present study, future studies on decreasing urea-formaldehyde (UF) resin content, or even using an eco-friendly resin within a green framework, would be predictable and should be carried out, similar to what was previously achieved by the application of tannin in wood-composite panels [27,28,29,30,31,32].

At the same time, Iranian wood-composite manufacturing factories confront the problem of shortages in wood fiber or particle resources to maintain sustainable production, therefore, potential natural fibers should be considered in order to meet the constant need for raw materials. In this way, numerous chicken farms exist in Iran, and therefore a huge amount of chicken feathers are in stock. It is reported by the Ministry of Agriculture of Iran that the production of chicken feathers in 2012 was about 80,000 metric tons; this figure corresponds to a manufacture of approximately 20 million composite panels by incorporating 5% feather content in to the wood furnishes. It also has to be mentioned that two million metric tons of chicken feathers are produced annually in the United States [26,27,28,29,30,31,32,33] whereas a figure of 3.1 million tons of feather waste is reported for the European Union [34,35,36]. Nowadays, high amounts of chicken feathers are disposed of in landfills and only a very small portion are converted into low-nutritional-value animal food [34,35,36]. This solution does not utilize the potential that this neglected material possesses, and more importantly, the management of environmental and health concerns becomes more difficult as overall waste rises. As chicken feathers are considered a waste raw material, it may be a cheap and renewable source for wood-composite industry. 

It is reported that chicken feathers were used as a reinforcement in manufacturing wood-cement composites, however no improvement in physicomechanical properties was found [37]. It is known that feathers consist of half quill and half fiber, by weight in approximate [33], which in turn consists of the hydrophobic protein keratin, which presents strength similar to that of nylon with a diameter smaller than that of the wood fiber. It is also worth mentioning that its covalent bonds stabilize the three-dimensional protein structure that is hard to break [38,39,40].

In the present study, chicken feathers were applied to the mat at a 5%- and 10%-dry-weight basis of wood fibers in the present research project. This approach would contribute to a more efficient use of natural resources and take advantage of this material that is produced in huge amounts and is currently underutilized by the poultry industry. It is intended that the use of materials from renewable resources contribute to sustainability and a reduction in the environmental impact associated with the incineration or disposing of poultry feathers into landfills. They are cheap, low density, abundantly available and renewable, delivering strong and stiff fibers, intrinsic characteristics of vital importance for the valorization of this waste for reinforcing material in composite materials. 

The present study was, therefore, primarily carried out to find a new source of natural fibers to feed the MDF-manufacturing factories in Iran which are greatly suffering from a shortage of raw materials (natural wood fibers). For this purpose, urea-formaldehyde resin (UF) was used because melamine-urea-formaldehyde (MUF) and other resins are neither popular in Iran’s market nor economical for the composite factories [6,20,21]. It should also be noted that the hydroxyl-groups of serine amino acids in feather fibers could possibly bind to wood fibers, contributing to the physical and mechanical properties of the MDF panels produced [24,33]. 

The separation of quill and feather-fiber was estimated to be costly and no wood composite manufacturing factories in Iran could afford such extra expenses. Therefore, in this study, the whole feather (quills and feather-fibers together) was used so that any potential positive results could directly be used at industrial scale. However, the quills of the wing feathers are not flexible and they created major problems in the preliminary tests. Therefore, only the body feather of chickens were used in the present study, considering their flexibility and small size of quills.

## 2. Materials and Methods

### 2.1. Specimen Procurement 

Wood fibers were procured from Sanaye Choobe Khazar Company in Amol of Iran (MDF Caspian Khazar). The fibers consisted of a mixture of five species, namely beech (*Fagus orientalis*), alder (*Alnus glutinosa*), maple (*Acer hyrcanum*), hornbeam (*Carpinus betulus*) and poplar (mostly *Populus nigra*) species from local forests (Amol, Iran). The target board thickness was 16 mm and the target density was 0.67 g/cm^3^. The temperature and the total nominal pressure of the plates were 175 °C and 160 bars respectively, whereas the press time was six minutes. Urea-formaldehyde resin (UF) was procured from Pars Chemical Industries Company, Tehran, Iran. UF content was 10% with 200–400 cP in viscosity, 47 s of gel time, and 1.277 g/cm^3^ in density. Produced panels were conditioned (25 °C, and 40% ± 3% relative humidity) for three weeks before testing. The moisture content of the board specimens at the time of testing was 7.5%. Five replicate panels were produced for each treatment. The board manufacture parameters are summarized in Table 1.

Wood chips were procured from Shahid Dr. Bahonar Composite-board Company (Gorgan, Iran) to produce particleboards. The chips comprised the same species as were used for wood fibers mentioned above; only a 5%–7% pruning branches of the fruit gardens was added. Boards were 16 mm in thickness and 0.67 g/cm^3^ in density; density was kept constant for all treatments. The same resin and production conditions were used in particleboard manufacturing program. Five boards were made for each treatment. 

**Figure 1:**
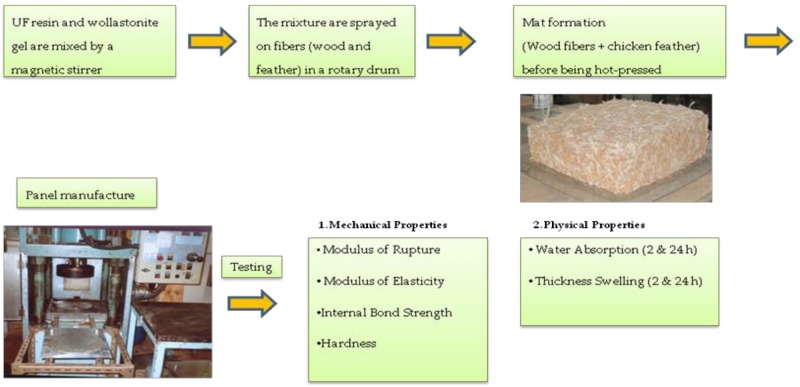
Flow diagram of the experimental procedure.

Feathers were procured from a commercial chicken farm located in Tehran, Iran. The preliminary evaluation of the costs revealed that separation of feather fibers from the quills would be costly and not encouraging for composite-manufacturing factories. It was decided that the whole feather would be used in this study so that any possible positive results could be directly used on a commercial scale. Therefore, only the feathers of the body, which are small and flexible enough for MDF production, were mixed with the wood fibers and chips in a drum-mixer to form the wood–chicken feather composite-mat. The length of the feathers ranged from one to three centimeters. The flow diagram of this experimental procedure is presented in Figure 1.

### 2.2. Wollastonite Application 

Wollastonite gel was produced in close cooperation with Mehrabadi Manufacturing Company in Tehran, Iran. Chemical composition of wollastonite used in the present study is presented in Table 2. More than 90% of wollastonite particles ranged 1–4 μm in thickness and width, and 5–25 μm in length. A total of 10% of wollastonite gel was applied, based on the dry weight of resin. Wollastonite was mixed with the UF resin by a magnetic stirrer for 20 min. The mixture of UF + Wollastonite was sprayed on fibers in a rotary drum. 

### 2.3. Temperature Measurement 

A digital thermometer with a sensor probe was applied in order to measure the temperature, with 0.1 °C precision, at the core of the mat, at 5-s intervals (Figure S1, from the Supplementary Materilas). Its 4-mm diameter probe was directly inserted into the core of the mat (from the front edge boarder of the mat), in horizontal direction, for about 50 mm. Temperature measurement was started immediately after the two hot plates reached the stop-bars. 

### 2.4. Physical and Mechanical Properties

Physical and mechanical properties were determined in accordance with the Iranian National Standard ISIRI 9044 PB Type P2 [41] (compatible with ASTM D1037-99) specifications. Mechanical properties were measured, using an INSTRON 4486 test machine. The physical properties included water absorption (WA) and thickness swelling (TS), after 2 and 24 h immersion in water. A digital scale with a 0.01 g precision was used for WA measurement. A digital caliper with a 0.01 mm precision was used for TS measurement. Five mechanical properties were also measured, including modulus of rupture, modulus of elasticity, brittleness, internal bond and hardness at 5.4 mm of penetration. Dimension of the specimens for physical properties (water absorption and thickness swelling), as well as internal bond test, were 50 mm × 50 mm. Dimension of modulus of rupture (MOR) and modulus of elasticity (MOE) specimens was 350 mm × 50 mm; the loading span was 320 mm. Specimens were loaded at a rate of three mm per minute. Brittleness was calculated based on Equation (1), in which the ratio (%) of the work absorbed in the elastic region divided by the total absorbed work is measured [42,43]. Once internal bond specimens were cut, the two faces in each of the test specimens were glued to an aluminum block, using hot-melt adhesive. The blocks were then pulled until failure. For hardness measurement, two specimens of 75 mm × 50 mm, each with a thickness of 16 mm, were bound together to prepare thickness of 32 mm according to the standard specifications. Hardness was then measured using a 11.28 mm diameter modified Janka ball, with a projected impact area of 100 mm^2^.

(1) $$\mathrm{Brittleness}=\frac{Area\;1}{Area\;1\;\;+Area\;2}\;\times \;100\;(\%)$$

### 2.5. Density Functional Theory

For a better understanding of how wollastonite reacted with carbohydrates in wood polymers, some simulations were performed. All simulations were carried out based on density functional theory (DFT) using the OpenMX3.8 package [25,26]. The exchange and correlation potential was described with generalized gradient approximation (GGA) of Perdew–Burke–Ernzerhof (PBE). The long-range Van der Waals interactions were included in the simulations by the DFT-D2 approach. The plane wave cutoff energy was uniformly set to 50 Ry in all three cell wall polymer calculations (cellulose, hemicellulose and lignin). 

Adsorption energy, *E*_ads_, was calculated by the Equation (2) in order to evaluate the interaction between wollastonite and hemicellulose or lignin,

*E*_ads_ = *E*_hemicellulose/lignin + Wollastonite_ − (*E*_hemicellulose/lignin_ + *E*_Wollastonite_) (2)

where *E*_hemicellulose/lignin + Wollastonite_ is the total energy of hemicellulose or lignin with adsorbed W (wollastonite); *E*_hemicellulose/lignin_ denotes the total energy of isolated hemicellulose or lignin; and *E*_Wollastonite_ is the total energy of the isolated W. The negative adsorption energy represents the stable adsorption structure.

### 2.6. Modelling of Wollastonite

Wollastonite crystals contain silicate chains along with the chain axis, linked to a periodicity of three tetrahedral. The calcium is linked by irregular octahedral coordination to six of the oxygen [25,26]. 

### 2.7. Modelling of Hemicellulose

Hemicellulose is a branched polysaccharide consisting of shorter chains of around 200 sugar units. Twenty percent of the biomass contains hemicellulose molecules derived from different sugar monomers like glucose, xylose, mannose, galactose, rhamnose, and arabinose [44]. The model of hemicellulose introduced by Kaith et al. [44] was elaborated in the present project to evaluate the adsorption of wollastonite and water molecules. 

### 2.8. Modelling of Lignin

Lignin is known as the second most abundant biopolymer on earth. It possesses a high content of aromatic groups. There are three monolignol building blocks in lignin, methoxylated to various degrees: p-coumaryl alcohol, coniferyl alcohol and sinapyl alcohol. These building blocks are incorporated into lignin in the form of the phenyl propanoids derivatives. In the present study, modeling was separately completed based on all three monolignols. This biopolymer contains small amounts of incomplete and modified monolignols, as well as other monomers. There is a wide range of different functional groups in lignin molecules, including aliphatic and aromatic hydroxyl groups, double bonds and phenyl groups [45].

### 2.9. Statistical Analysis

SAS software program was used to carry out statistical analysis in the present study (version 9.2; 2010, SAS Institute Inc., Cary, NC, USA). To discern significant difference among different treatments and produced panels, one-way analysis of variance was performed at 95% level of confidence. Then, Duncan’s multiple range test (DMRT) was completed to group each property among treatments. In order to find degrees of similarities among different treatments based on all properties studied here, hierarchical cluster analysis from SPSS/18 (2010) software was used. For graphical statistics (fitted-line, contour and surface plots), Minitab software was utilized (version 16.2.2; 2010, Minitab Inc., State College, PA, USA). 

## 3. Results and Discussion

### 3.1. Temperature of the Core of Composite Mats

**Figure 2:**
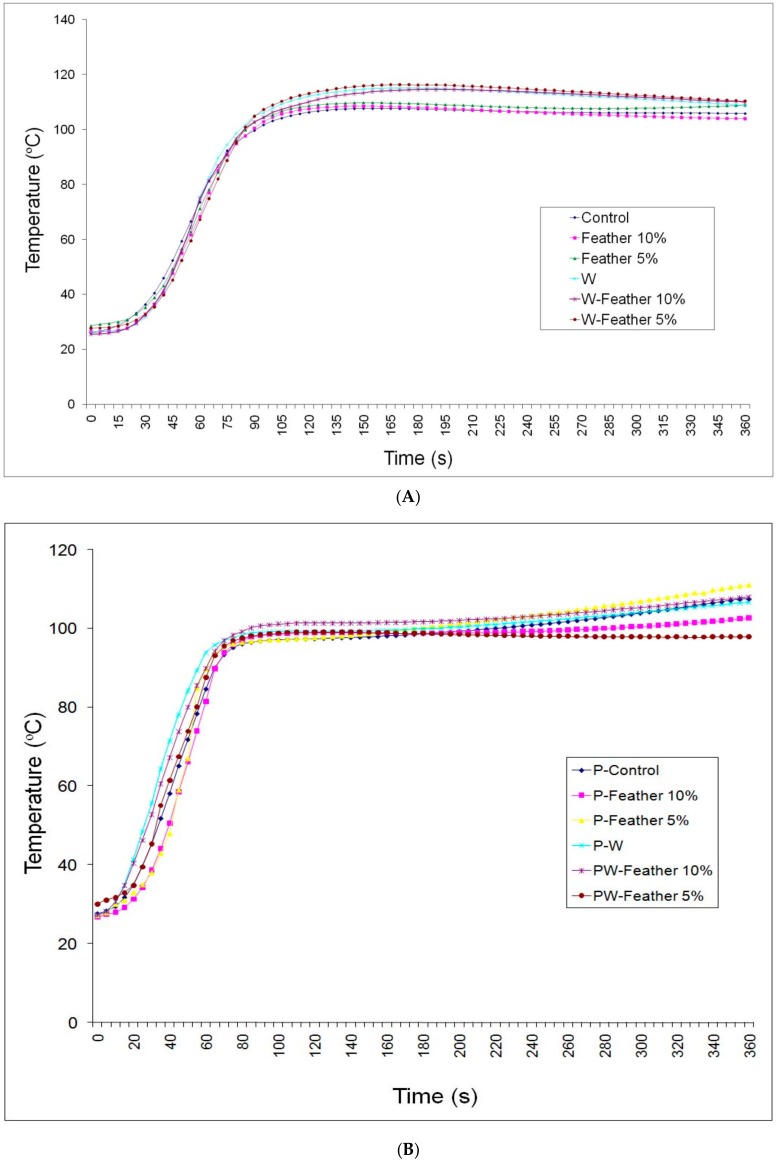
Temperature (Celsius) at the core section of the MDF (**A**), and the particleboard (**B**) at 5-s intervals (P = particleboard; MDF = medium-density fiberboard; W = wollastonite; S = time intervals).

Measurement of temperature at the core of composite mats revealed a significant difference between MDF panels without wollastonite and the three wollastonite-treated panels (Figure 2A). All treatments showed an almost identical increase up to 90 s; however, the three wollastonite-treated panels showed a clear higher temperature after the first 90 s. This clearly showed the effects of the higher thermal conductivity coefficient in wollastonite-treated panels on the heat transfer to the core section of the composite mat [20].

Measurement of the core section of the particleboard mats showed a significant lower temperature in comparison to the MDF mats (Figure 2B). This can be attributed to the higher contact surface among wood fibers (MDF matrix) in comparison to wood particles (particleboard matrix); that is, the surface-to-surface contact is higher between wood fibers in comparison to the contact between wood particles, so the heat of the hot-press plates could more rapidly be transferred to the core section in MDF mat. 

### 3.2. Physical Properties

Water absorption (WA) was the same in the three MDF-treatments without wollastonite, both for 2 and 24 h immersion in water (Figure S2, from the Supplementary Materials). This showed that addition of feather to the MDF-matrix did not significantly affect the water absorption. Wollastonite-treated panels showed a significant decrease in water absorption for all the three treatments. It was previously reported that wollastonite-treated composite panels had lower gas and liquid permeability [20,26]. In this way, the reinforcement of UF resin by wollastonite caused higher integration of fiber in the composite-matrix, preventing water to easily pass through. Similar reinforcement in resin and paint was previously reported by the addition of wollastonite and graphene [15]. Moreover, the formation of bonds between wollastonite and wood polymers prevented wood hydroxyl groups to be actively involved in making bonds with water molecules [25,26], decreasing WA in all treatments. 

In particleboards specimens, the procedure was somehow different; wollastonite decreased water absorption only after 24 h immersion in water (Figure S2, from the Supplementary Materials). The addition of feathers (both 5% and 10% contents) significantly increased water absorption after 2 h of immersion, and wollastonite could not compensate for it, probably because chicken feathers reached their maximum moisture content. However, wollastonite could control WA to some extent after 24-h immersion.

The lowest thickness swelling occurred in wollastonite-treated 5%-feather content MDF panels. The addition of wollastonite or feathers at both 5% and 10% contents generally resulted in a significant decrease in thickness swelling in MDF panels after both 2 and 24 h immersion in water (Figure S3, from the Supplementary Materials). This was attributed to the reinforcing effect of wollastonite and formation of bonds between wollastonite and wood polymers; moreover, the hydrophobic properties of keratin in feathers contributed to this phenomenon. 

In particleboard panels, the procedure was different again (Figure S3, from the Supplementary Materials). The highest thickness swelling occurred in 10%-feather content particleboard panels with no wollastonite content. In fact, lower surface-to-surface contact between wood-feather-matrix as well as the voids and spaces between the wood particles provided more opportunity for water to penetrate into the particleboard-matrix; however, by addition of wollastonite to panels, water penetration could be controlled significantly in 10%-feather content wollastonite-panels. In the meantime, the addition of 5% feathers could even improve thickness swelling at 2-h immersion. The lowest thickness swelling values were found in wollastonite-treated particleboard panels.

### 3.3. Mechanical Properties

**Figure 3:**
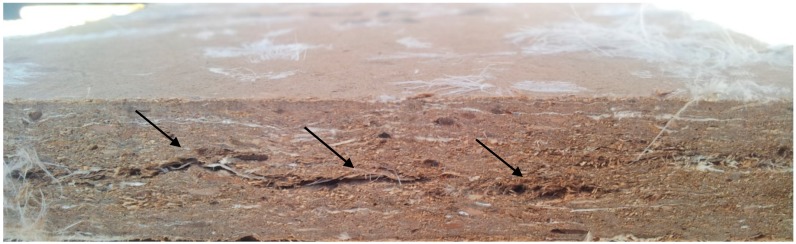
Cracks (blows) (↓) in the core layer of MDF-feather boards (F-10%).

The highest modulus of rupture was observed in wollastonite-treated MDF panels without feathers (15.5 MPa) (Figure S4, from the Supplementary Materials). Wollastonite improved modulus of rupture in all panels. This improvement was attributed to reinforcement of resin [15,46], as well as formation of new bonds between wollastonite and wood polymers [25,26]. The addition of feathers to the MDF panels significantly decreased modulus of rupture. In this connection, the level of decrease in 5%-feather content was small in comparison to the high significant decrease that occurred in the 10%-feather content panels. In fact, visible checks and cracks (internal blows) occurred in the core section of the mat in 10%-feather MDF panels (Figure 3). This clearly showed that this amount of feather content would not be suitable for MDP panels made with UF resin, as the UF resin is not compatible with keratin in chicken feathers. These cracks were reported to be the main reason for the higher mass loss values in specimens exposed to fungi attack [24]. It was concluded that 10%-feather content was too high, but 5%-feather could be considered suitable to satisfy the fiber shortage and keep the physical and MOR properties at satisfactory level. The addition of wollastonite decreased MOR in particleboards (Figure S4, from the Supplementary Materials). Only panels with 10% feather content showed an improvement by addition of wollastonite, maybe due to the higher hygroscopicity of chicken feather.

The highest modulus of elasticity was found in wollastonite-treated MDF panels without feathers (1760 MPa) (Figure S5, from the Supplementary Materials). Panels with 5%-feather content showed significant increase in modulus of elasticity in comparison to panels with no feather, proving the elastic-increasing effect of feathers in the MDF-matrix. However, the 10%-feather content seemed to be too high and resulted in a significant decrease in MOE. The addition of wollastonite to panels manufactured with 10% feather content could improve MOE to as high as that of control panels. An almost identical trend was seen in particleboard panels; the highest MOE value was observed in wollastonite-treated panels without feathers, and the addition of feathers to the matrix significantly decreased modulus of elasticity (Figure S5, from the supplementary materials). 

Brittleness was not significantly changed with either addition of wollastonite or feathers at 5% consumption level in MDF panels (Figure S6, from the Supplementary Materials). However, the addition of the 10%-feather caused a significant increase in brittleness in MDF panels. This again showed that the 10%-feather content was too high. Wollastonite clearly decreased brittleness in all three particleboard treatments (control, 5%- and 10%-feather contents) (Figure S6, from the Supplementary Materials). The addition of wollastonite to the wood fibers or wood particles significantly decreased internal bond in the MDF and particleboard panels without feather content (Figure S7, from the Supplementary Materials). This was attributed to the absorption or gathering of resin molecules by wollastonite particles, preventing them from being active in the process of sticking the strips together. Moreover, acting as a kind of filler, wollastonite improved modulus of rupture and hardness. However, the measurement of internal bond requires resins to be under pulling force. Wollastonite did not have improving effect on the pulling force of UF resin. 

Hardness was measured at 3, 4, 5 and 5.4 mm penetration depths in order to gain a better understanding of the effects of the addition of wollastonite and feathers on the surface or inner parts of panels. Almost identical trends in increase and decrease were observed in all four depths of penetration of the steel ball in both MDF and particleboard panels, indicating that the effects were the same at different depths (Figure S8, from the Supplementary Materials). Feathers significantly decreased hardness in both MDF and particleboard panels, which were quite predictable due to the softness of feathers in comparison to wood fibers and particles. Furthermore, the particleboard panels showed significantly higher hardness values in comparison to MDF panels. The addition of wollastonite increased hardness in MDF-feather panels, although not significantly in 5%-feather content panels. However, in particleboard panels, no significant trend was observed. 

### 3.4. Adsorption of Wollastonite on Wood Cell Wall Polymers

Different configurations of wollastonite on hemicellulose were constructed by floating and rotating wollastonite on the surface of hemicellulose and lignin molecules. First, wollastonite was placed far away from the cellulose or lignin surfaces. Then, the distance of wollastonite from the surface was gradually decreased so that the optimal adsorption distance was found based on minimum adsorption energy. The closest distance between wollastonite and the hemicellulose surface, and the adsorption energy of the most stable structure, were found to be 1.7 Å and −4.5 eV, respectively. This large adsorption energy revealed a strong adsorption of wollastonite on hemicellulose as a result of the formation of bonds between them, which proved to be effective in holding wollastonite on the hemicellulose surface.

The comparison between adsorption energies of wollastonite on cellulose and hemicellulose demonstrated a stronger energy bond between wollastonite and cellulose. The adsorption energies of wollastonite on cellulose and hemicellulose were −6.6 and −4.5 (eV), respectively. The stronger bond with cellulose was mainly attributed to the fact that cellulose is a straight chain polymer with no branches; consequently, it provided smoother surface for wollastonite to be easily adsorbed on it. However, hemicellulose is a branched chain polymer; the branches made it difficult for the wollastonite to make bonds with it. It should be noted that as far as water absorption is concerned, hemicellulose has a higher number of hydroxyl groups and is more hydrophilic in comparison to cellulose [47,48]. However, in regard to the adsorption energy of wollastonite on either cellulose or hemicellulose, a single bond between wollastonite and cellulose has more energy than that of hemicellulose.

Water molecules with various orientations were placed on different functional groups of hemicellulose to clarify adsorption of water on hemicellulose. The results showed formation of hydrogen bonds between water molecules and hemicellulose (OH_water_…O_hemicellulose_ and OH_water_…OH_hemicellulose_). OH_water_ indicated the hydroxyl group of adsorbed water molecule; O_hemicellulose_ and OH_hemicellulose_ represented hydroxyl groups of hemicellulose, respectively. 

Though wollastonite demonstrated higher adsorption energy in comparison to water molecules, the increase of the number of adsorbed water molecules to twelve molecules gave a competition priority to water over W. 

The calculated adsorption distance between wollastonite and lignin was 1.8 Å, and the most stable structures had an average adsorption energy of −2.6 eV. The large adsorption distance along with the small adsorption energy indicated that the adsorption of wollastonite on lignin was so weak that it can practically be ignored. This is quite consistent with the fact that lignin is considered a hydrophobic element [47,48]. 

Moreover, the adsorption of one water molecule was separately investigated on three different monolignols of lignin (namely, p-coumaryl alcohol, coniferyl alcohol and sinapyl alcohol). Adsorption energies of all three alcohols were positive. The positive energy is considered corroborating evidence of the hydrophobicity of lignin, implying than none of the three structures were energetically stable and, therefore, water molecules could not practically be adsorbed on them. This can be explained by the fact that hydrogen bonds between water and lignin cannot be formed because of the lack of hydroxyl groups in lignin. Ultimately, lignin is hydrophobic in nature. Still, it should be noted that the main reason for the hydrophobic nature of lignin could be the existence of more phenolic groups in its structure. 

### 3.5. Relation between Physical and Mechanical Properties

A fitted-line plot between MOR versus MOE revealed a significant relation (R-square of 100%). This showed the direct effect of an increase or decrease in one property on the other. In addition, a high significant R-square was found between MOR versus brittleness and hardness, although not as high as that in MOE. A low R-square (63%) was found between MOR versus internal bond. With due consideration to the fact that the four properties of MOR, MOE, hardness and brittleness are mostly dependent on the surface layers of specimens rather than the core section, the high significant correlations are justified. The internal bond, however, is mainly dependent on the properties of the core of the composite panels, showing that the properties of the surface layers and core layer of composite panels may be quite independent to each other.

**Figure 4:**
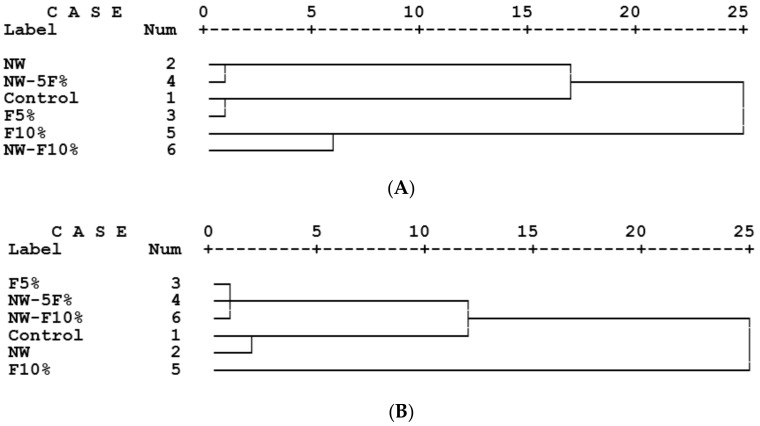
Cluster analysis in the medium-density fiberboard (**A**) and particleboard (**B**) panels based on all the physical and mechanical properties studied in this study (W = wollastonite; F = feather content).

The cluster analysis of the MDF panels based on all physicomechanical properties studied (water absorption and thickness swelling after 2 and 24 h immersion in water, modulus of rupture, modulus of elasticity, brittleness, internal bond and hardness at 5.4 mm of penetration) showed a different clustering of control and wollastonite-treated panels. The cluster analysis identified the significant effects of wollastonite on the overall physical and mechanical properties of medium-density fiberboards (Figure 4A). Wollastonite–5%-feather treatment was closely clustered to wollastonite-treated panels; this clearly showed that, although there was an addition of feathers to the MDF-matrix, and a significant diminishment in properties was anticipated, wollastonite could compensate for the loss to a great extent. With due consideration to the mitigating effects of wollastonite on the overall properties, future studies on decreasing resin content are to be carried out, similar to what was previously achieved by the application of tannin in wood-composite panels [27,28,29,30,31,32]. Moreover, 5%-feather treatment was closely clustered to the control panels; this indicated that through addition of 5% of feathers to the MDF-matrix, the overall properties remained the same. Therefore, it can be concluded that chicken feathers can be used in MDF manufacturing programs. However, the addition of 10% of feathers to the MDF-matrix resulted in a significant difference in the overall panel properties; 10%-feather panels were remotely clustered to the rest of the treatments. 

In particleboard panels, control panels (without wollastonite or feather content) were closely clustered to the wollastonite-treated panels (Figure 4B). This showed that wollastonite did not have significant effects on the overall physicomechanical properties. Panels with 10% feather content were remotely clustered with all the other treatments, showing that this feather content was not suitable for the production of particleboards. 

**Figure 5:**
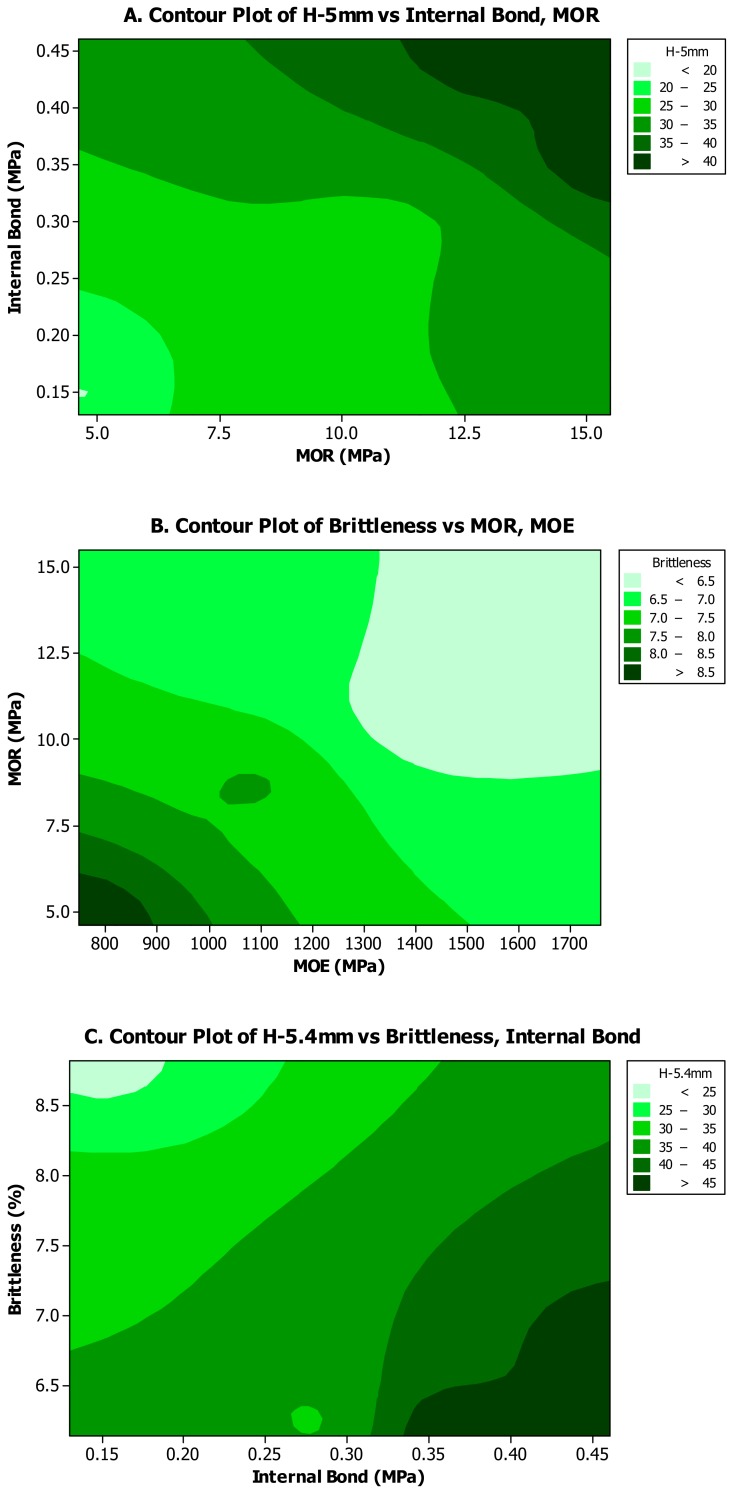

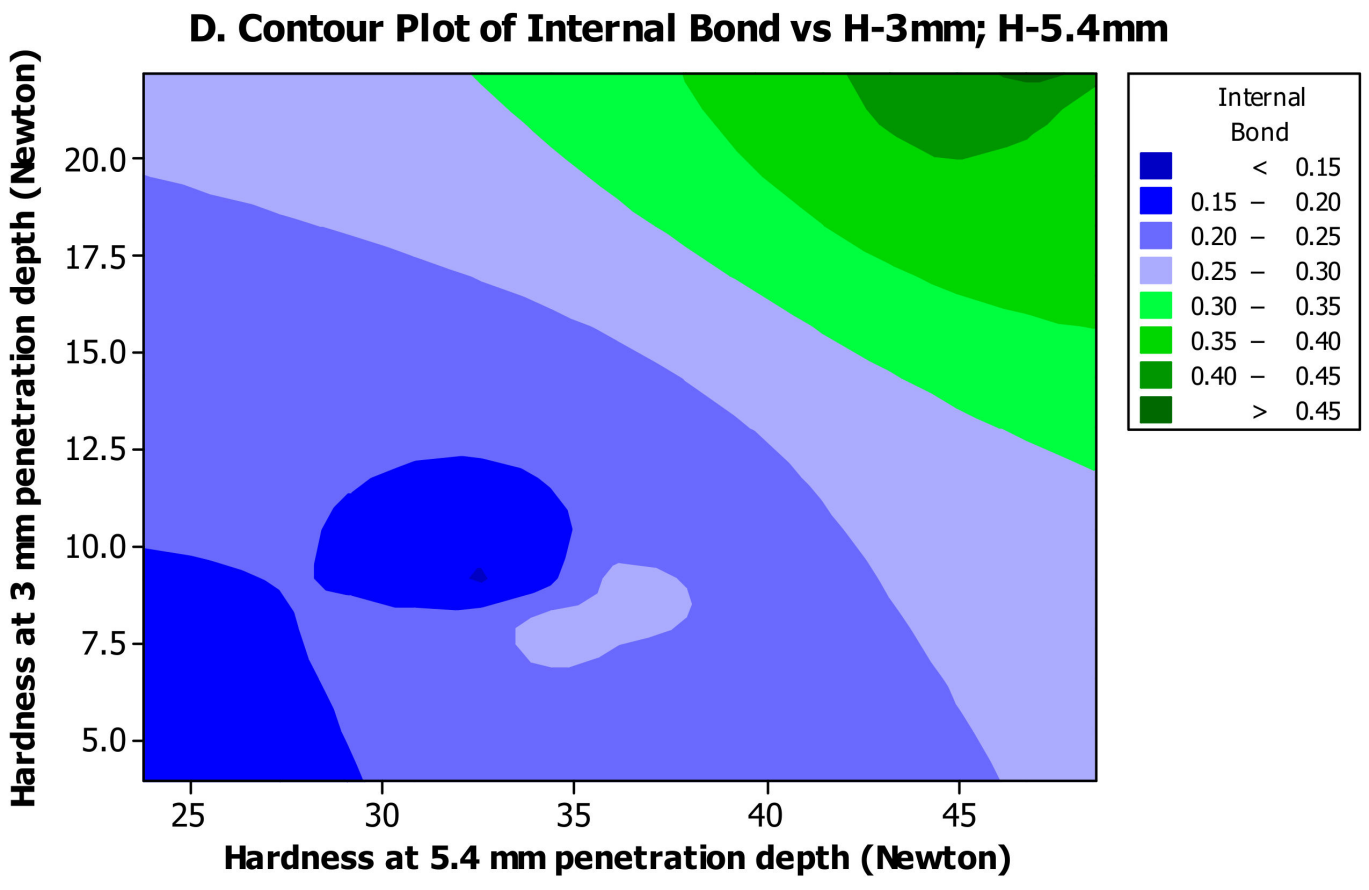
Contour plots among different properties of composite panels observed in this study. (**A**) among MOR and internal bond properties versus hardness at 5-mm penetration depth; (**B**) among MOR and MOE properties versus brittleness; (**C**) among brittleness and internal bond properties versus hardness at 5.4 mm penetration depth; (**D**) among hardness at 3 mm and 5.4 mm penetration depth versus internal bond. (MOR = modulus of rupture; MOE = modulus of elasticity).

Contour plots showed an increasing relationship of hardness values versus internal bond and MOR values (Figure 5A). However, brittleness showed a completely inverse relationship with the mechanical properties of MOR and MOE (Figure 5B). Moreover, it was found that hardness had a straight relationship with internal bond values, but an inverse relationship with brittleness (Figure 5C). The contour plot of internal bond versus hardness at two depths (3 and 5.4 mm) demonstrated a direct relationship with both shallow and deeper penetrations of the hardness ball (Figure 5D); this implied that addition of wollastonite and feathers to mat had similar effects on different layers of composite panels.

## 4. Conclusions

Chicken feathers were mixed at 5% and 10% consumption levels with wood fibers and particles to produce medium-density fiberboard (MDF) and particleboard panels, in order to comply with the growing need for new sources of raw materials. Urea-formaldehyde (UF) resin was used as the binder. Wollastonite was mixed in UF resin to mitigate the potential negative effects of chicken feathers, and also to investigate if the addition of wollastonite has any potential in future studies to decrease resin content in composite panels in the same way that tannin was reported. The addition of 10%-feather resulted in significant negative effects on all physical and mechanical properties. A feather content of 5% showed some promising results. Wollastonite acted as reinforcing filler in the resin, improving most of the physical and mechanical properties. It was concluded that chicken feathers have potential in wood-composite production.

## Figures and Tables

**Table 1:** Board manufacture parameters.

**Board Density**	0.67 g/cm^3^
**Board Thickness**	16 mm
**Press Temperature**	175 °C
**Press Time**	6 min
**Pressure of Plates**	160 bars
**Resin Type and Content**	10% urea formaldehyde (UF) resin
**Resin Characteristics**	200–400 cP in viscosity, 47 s of gel time, and 1.277 g/cm^3^ in density.
**Wollastonite Content**	10% of UF resin (based on the dry weight of the resin)

**Table 2:** Composition of the wollastonite used in the present study.

Component	Proportion (% w/w)
SiO_2_	47.1
CaO	39.9
Al_2_O_3_	3.9
Fe_2_O_3_	2.8
TiO_2_	0.2
K_2_O	0.04
MgO	1.4
Na_2_O	0.2
SO_3_	0.05
Water	The rest

## Supplementary Materials

The following are available online at https://www.mdpi.com/2073-4360/12/4/857/s1, Figure S1: Temperature measurement at the core of the mat with 5-second intervals with a digital thermometer using a thermocouple probe inserted into the center of the core of the MDF-mat; Figure S2: Water absorption (%) in medium-density fiberboard (A) and particleboard (B) panels after 2 and 24 hours immersion in distilled water (MDF=medium-density fiberboard; PB = particleboard panels; NW=nano-wollastonite; WA = water absorption); Figure S3: Thickness swelling (%) in medium-density fiberboard (A) and particleboard (B) panels after 2 and 24 hours immersion in distilled water (MDF=medium-density fiberboard; PB = particleboard panels; NW=nano-wollastonite; TS = thickness swelling); Figure S4: Modulus of rupture (MPa) in medium-density fiberboard (A) and particleboard (B) panels (MDF=medium-density fiberboard; PB = particleboard panels; NW=nano-wollastonite); Figure S5: Modulus of elasticity (MPa) in medium-density fiberboard (A) and particleboard (B) panels (MDF=medium-density fiberboard; (PB = particleboard panels; NW=nano-wollastonite); Figure S6. Brittleness (%) in medium-density fiberboard (A) and particleboard (B) panels (MDF = medium-density fiberboard; PB = particleboard panels; NW = nano-wollastonite); Figure S7. Internal bond (MPa) in medium-density fiberboard (A) and particleboard (B) panels (MDF = medium-density fiberboard; PB = particleboard panels; NW = nano-wollastonite; Figure S8. Hardness (N) in medium-density fiberboard (A) and particleboard (B) panels after 3, 4, 5, and 5.4 mm of penetration into the MDF-matrix (MDF = medium-density fiberboard; PB = particleboard panels; NW = nano-wollastonite).
