# Supplementary material for: Engineering Composites Made from Wood and Chicken Feather Bonded with UF Resin Fortified with Wollastonite: A Novel Approach
Source: Polymers (Basel). 2020 Apr 7;12(4):857. doi: 10.3390/polym12040857 (PMC7240470; doi:10.3390/polym12040857)
Supplement: Supplementary file 1 [file polymers-12-00857-s001.pdf]

polymers

MDPI

## Supplementary Materials

# Engineering Composites Made from Wood and Chicken Feather Bonded with UF resin Fortified with Wollastonite: A Novel Approach

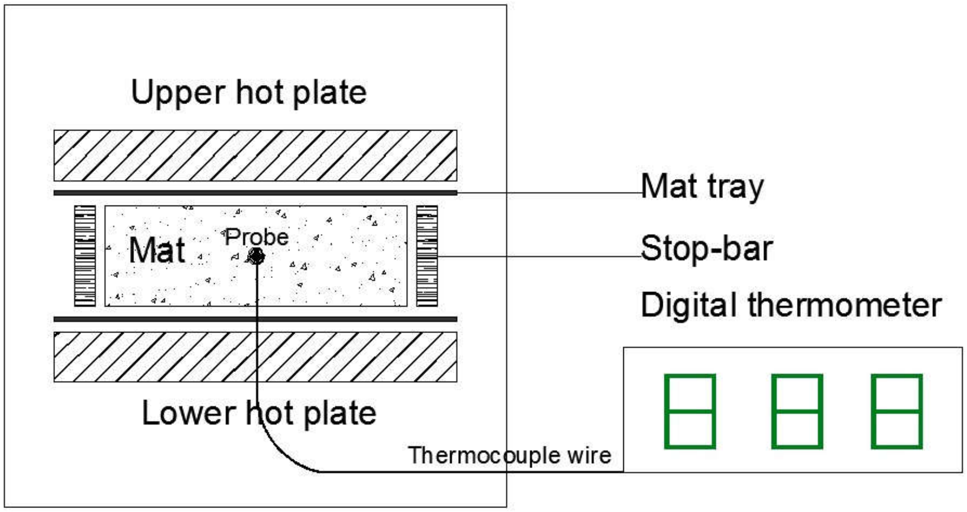


**Figure S1.** Temperature measurement at the core of the mat with 5-second intervals with a digital thermometer using a thermocouple probe inserted into the center of the core of the MDF-mat

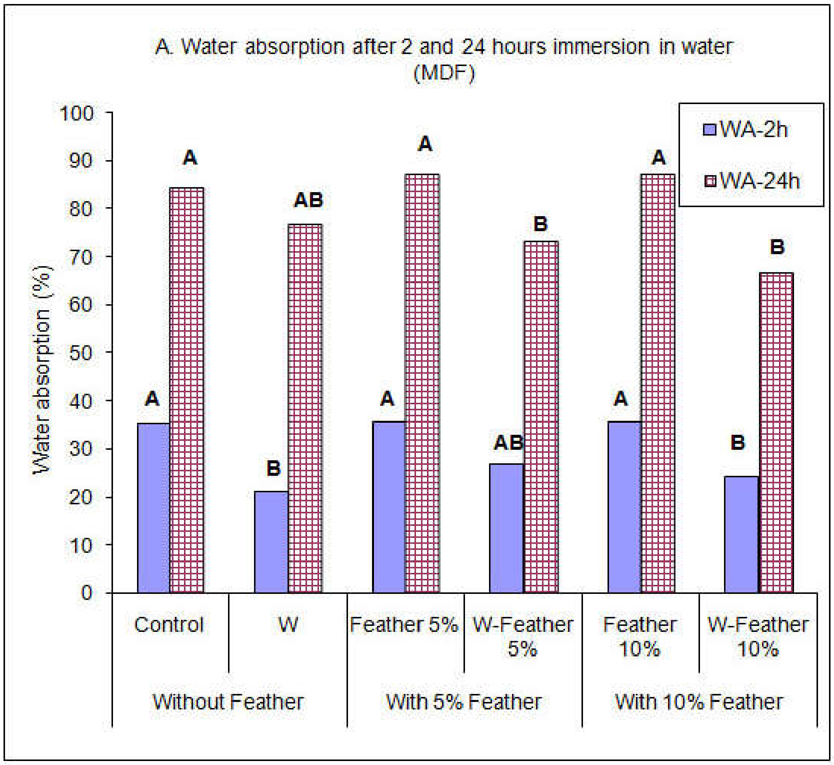


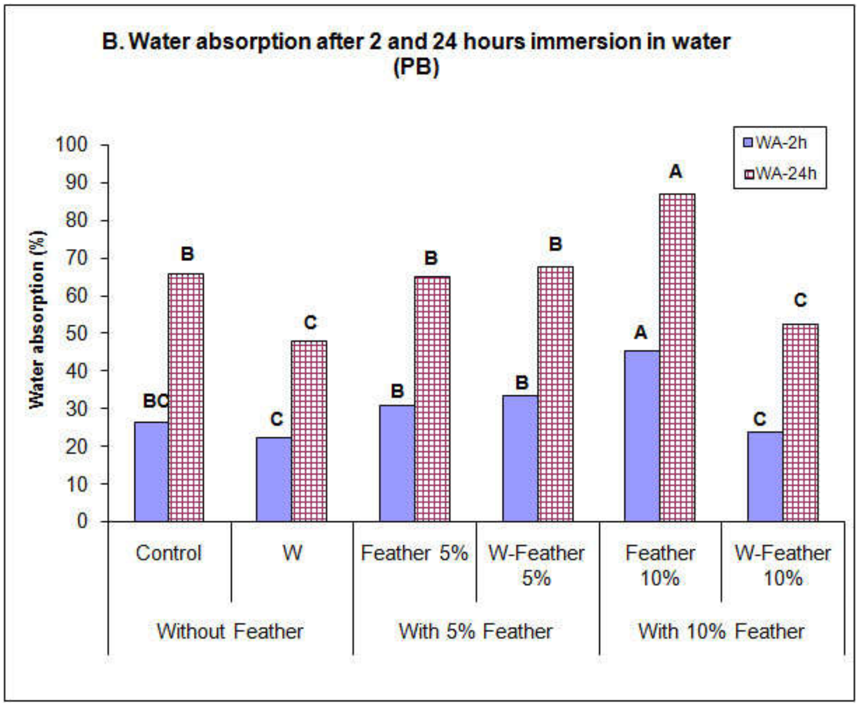


**Figure S2.** Water absorption (%) in medium-density fiberboard (**A**) and particleboard (**B**) panels after 2 and 24 hours immersion in distilled water (MDF=medium-density fiberboard; PB = particleboard panels; NW=nano-wollastonite; WA = water absorption).

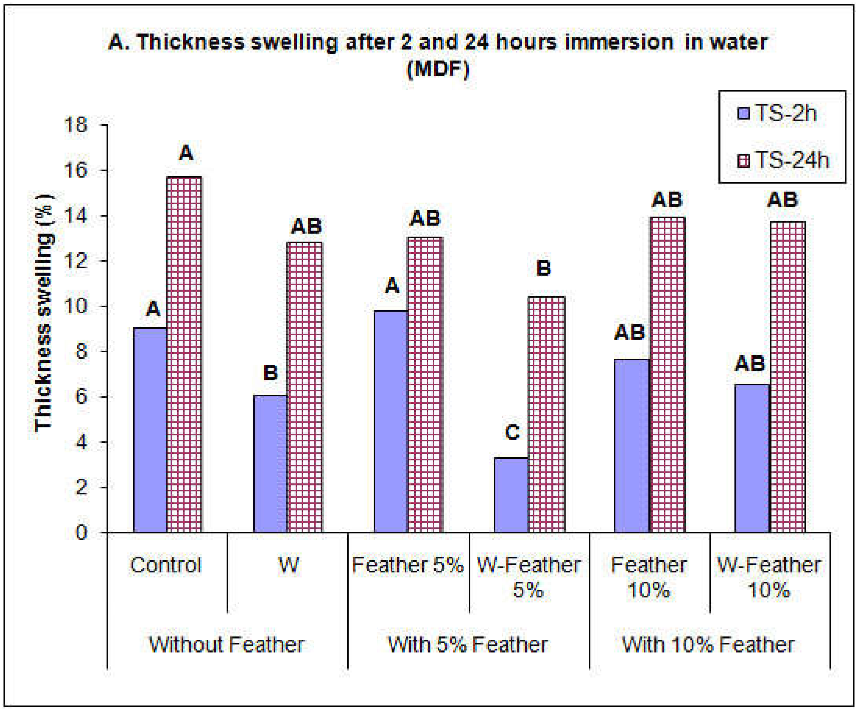


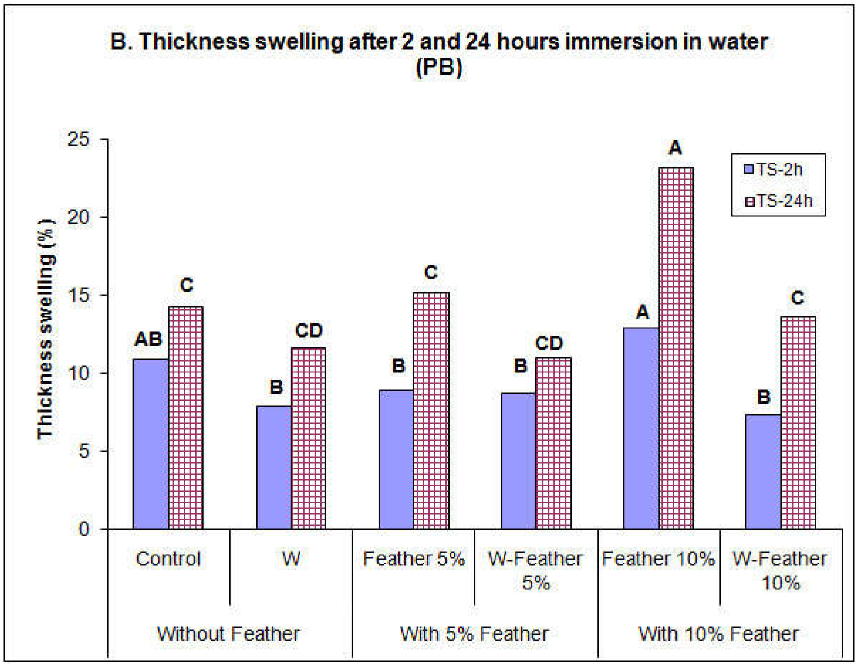


**Figure S3.** Thickness swelling (%) in medium-density fiberboard (**A**) and particleboard (**B**) panels after 2 and 24 hours immersion in distilled water (MDF=medium-density fiberboard; PB = particleboard panels; NW=nano-wollastonite; TS = thickness swelling)

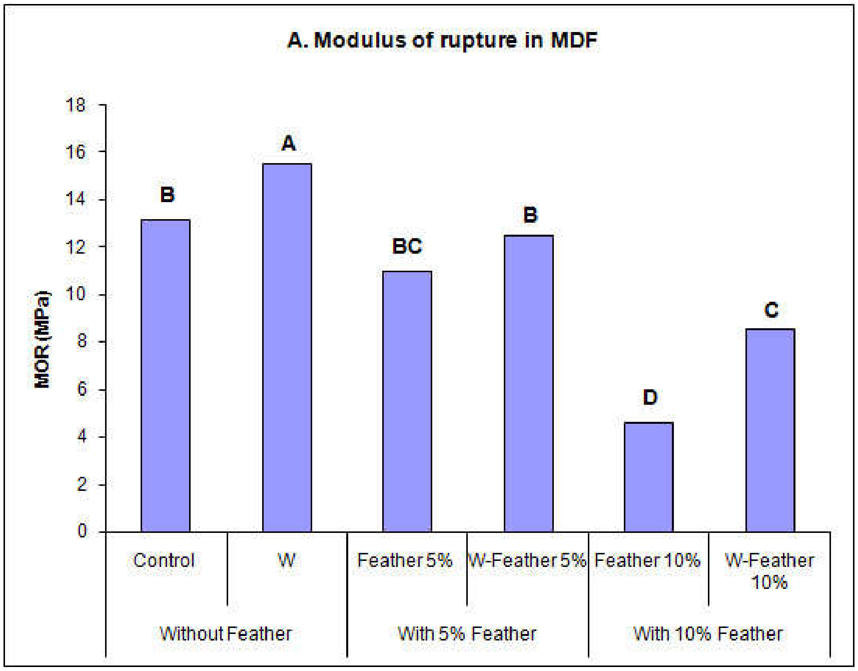


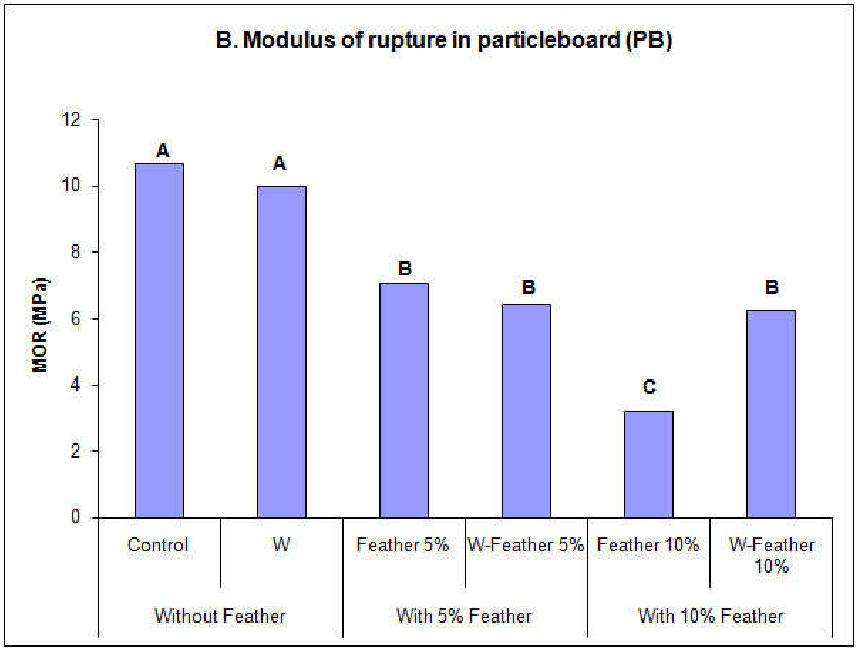


**Figure S4.** Modulus of rupture (MPa) in medium-density fiberboard (**A**) and particleboard (**B**) panels (MDF=medium-density fiberboard; PB = particleboard panels; NW=nano-wollastonite)

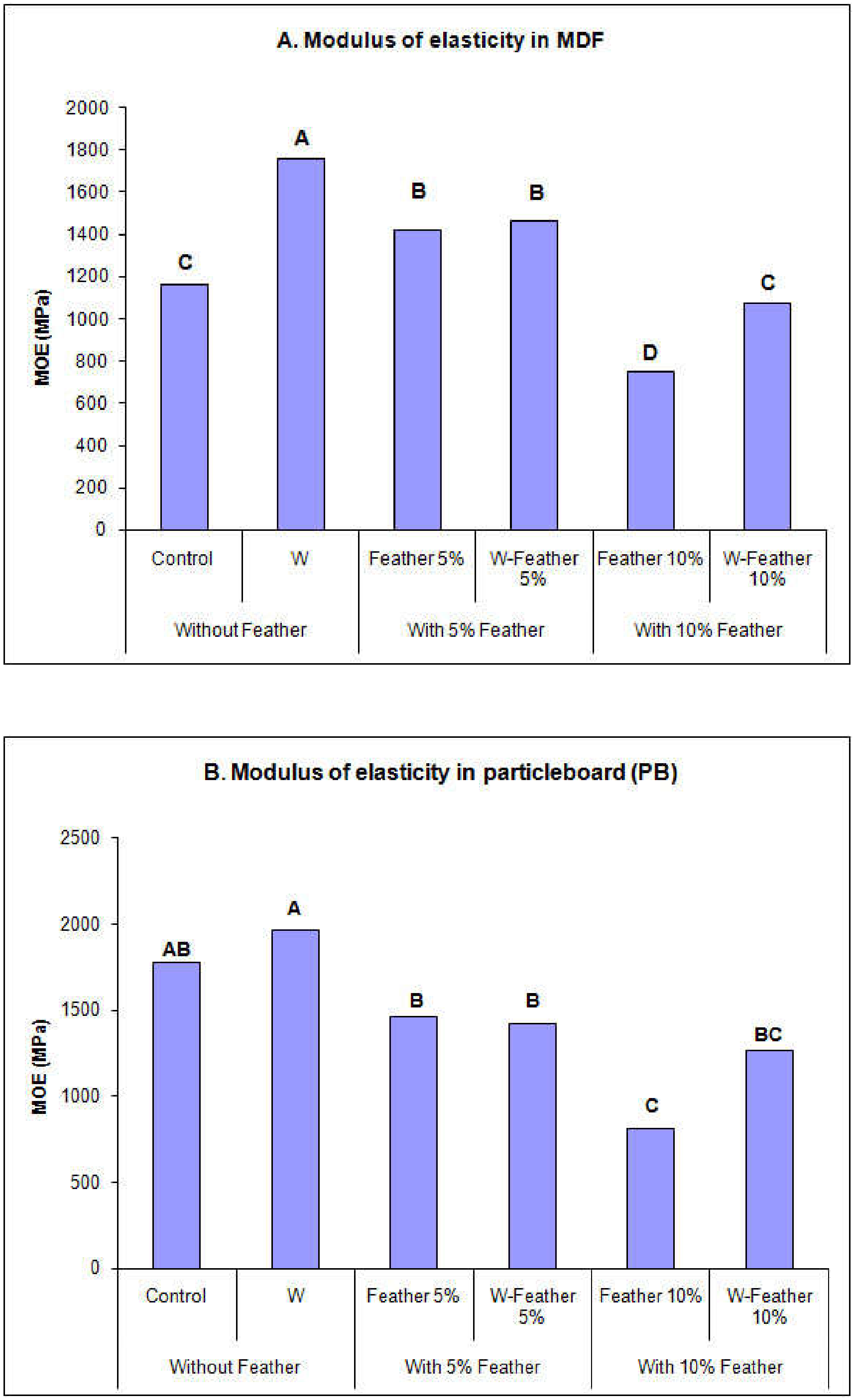


**Figure S5.** Modulus of elasticity (MPa) in medium-density fiberboard (**A**) and particleboard (**B**) panels (MDF=medium-density fiberboard; (PB = particleboard panels; NW=nano-wollastonite)

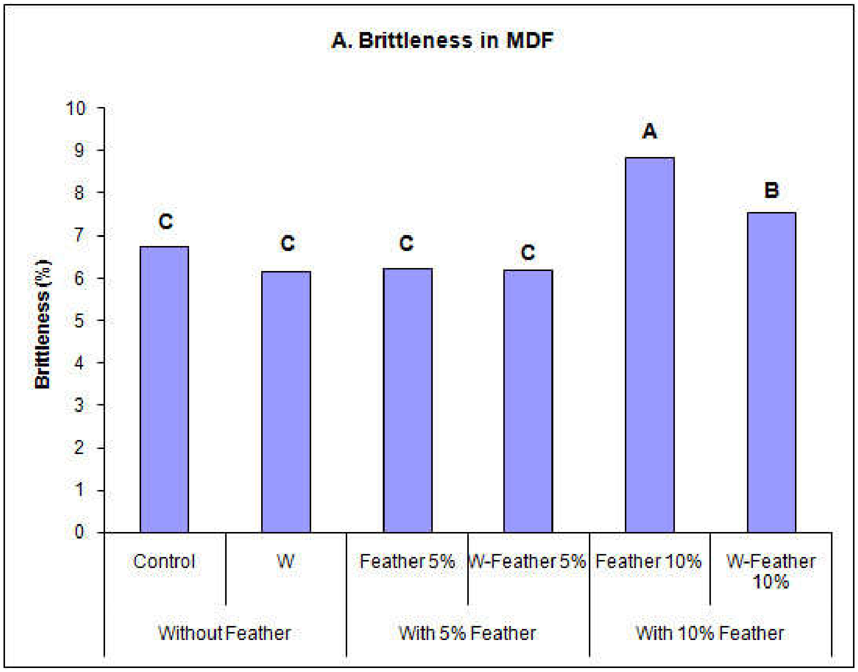


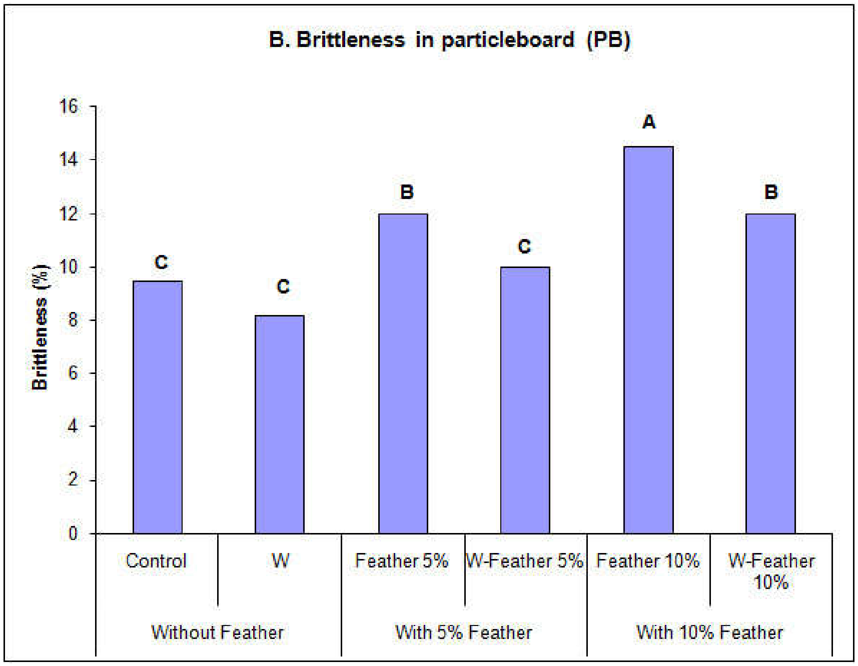


**Figure S6.** Brittleness (%) in medium-density fiberboard (**A**) and particleboard (**B**) panels (MDF = medium-density fiberboard; PB = particleboard panels; NW = nano-wollastonite)

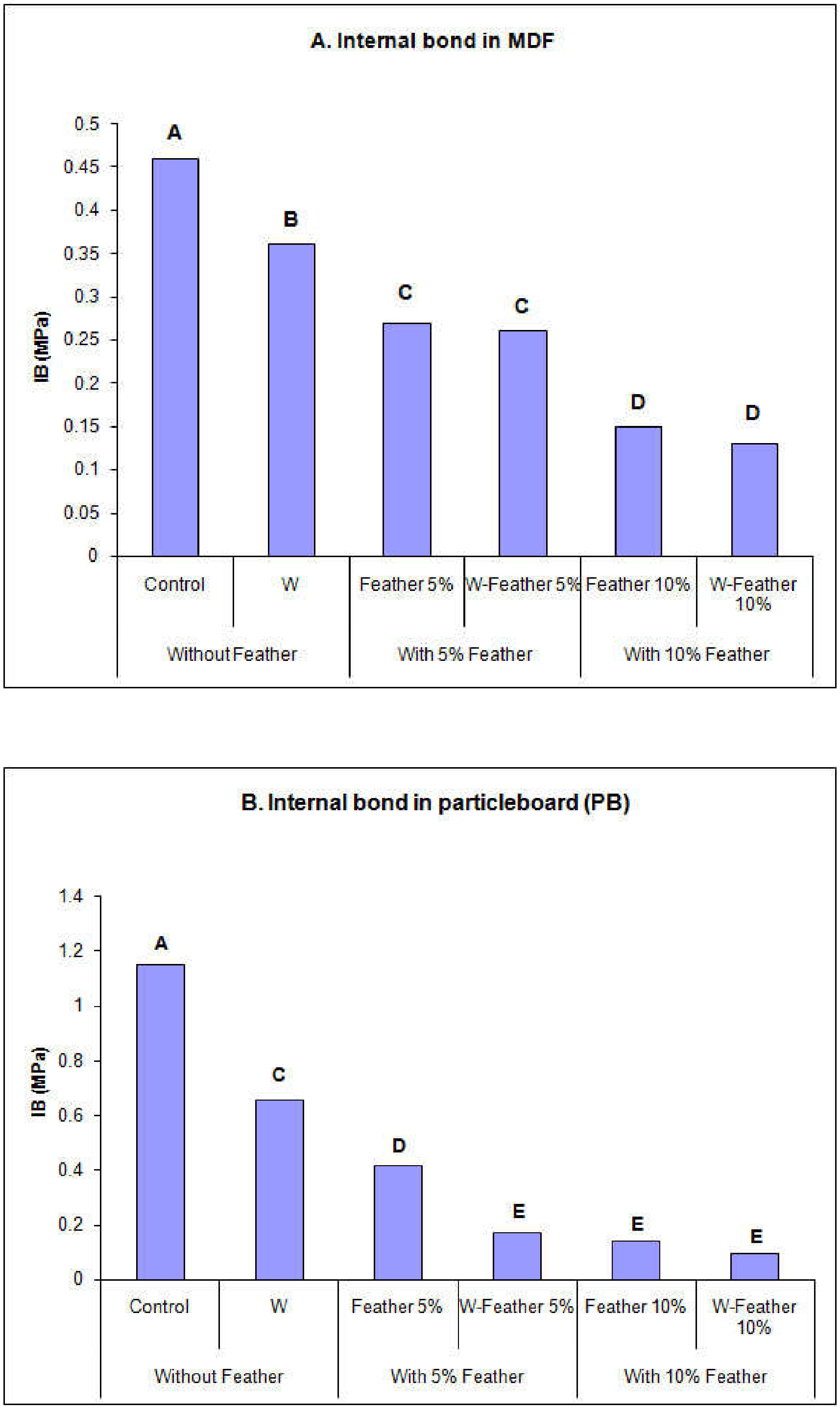


**Figure S7.** Internal bond (MPa) in medium-density fiberboard (**A**) and particleboard (**B**) panels (MDF = medium-density fiberboard; PB = particleboard panels; NW = nano-wollastonite)

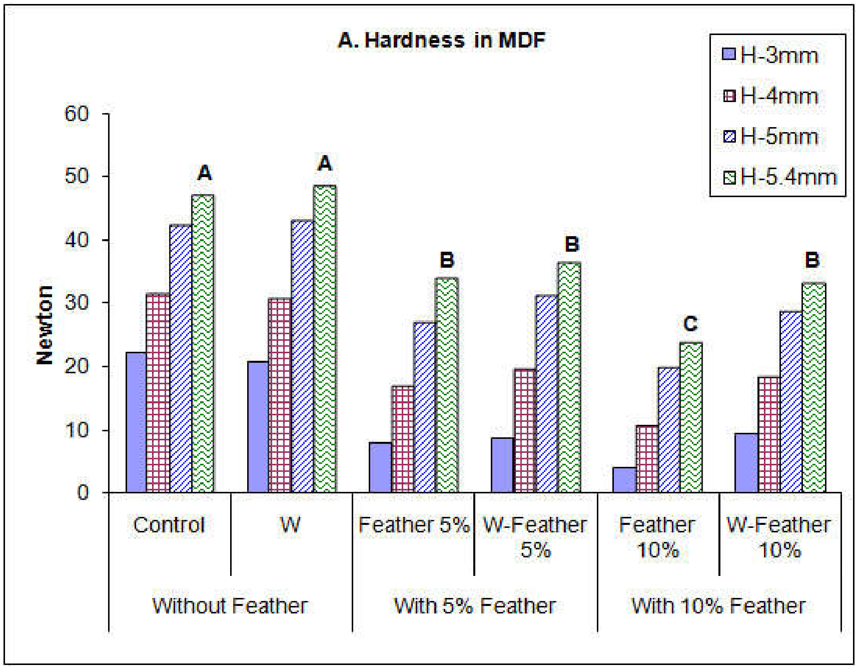


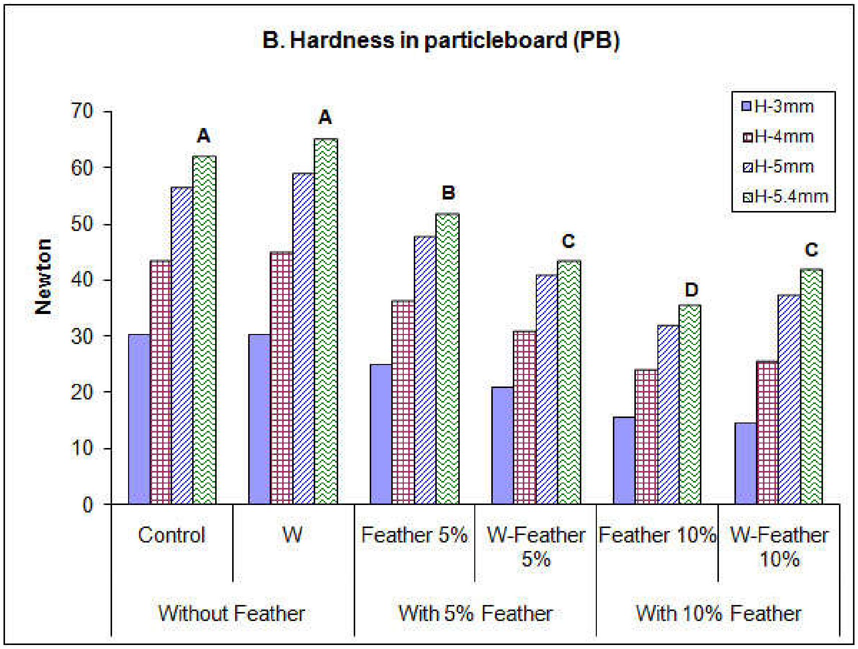


**Figure S8.** Hardness (N) in medium-density fiberboard (**A**) and particleboard (**B**) panels after 3, 4, 5, and 5.4 mm of penetration into the MDF-matrix (MDF = medium-density fiberboard; PB = particleboard panels; NW = nano-wollastonite)
